# Real-World Testing of a Machine Learning–Derived Visual Scale for Tc99m TRODAT-1 for Diagnosing Lewy Body Disease: Comparison with a Traditional Approach Using Semiquantification

**DOI:** 10.3390/jpm12091369

**Published:** 2022-08-25

**Authors:** Pai-Yi Chiu, Po-Nien Hou, Guang-Uei Hung, Te-Chun Hsieh, Pak-Ki Chan, Chia-Hung Kao

**Affiliations:** 1 Department of Neurology, Show Chwan Memorial Hospital, Changhua 50008, Taiwan; 2 Department of Applied Mathematics, Tunghai University, Taichung 40704, Taiwan; 3 Department of Nuclear Medicine, Chang Bing Show Chwan Memorial Hospital, Changhua 50544, Taiwan; 4 Department of Nuclear Medicine and PET Center, China Medical University Hospital, Taichung 40402, Taiwan; 5 Department of Biomedical Imaging and Radiological Science, Elite Campus, China Medical University, Taichung 40402, Taiwan; 6 Center of Augmented Intelligence in Healthcare, China Medical University Hospital, Taichung 40402, Taiwan; 7 Graduate Institute of Biomedical Sciences, Elite Campus, School of Medicine, College of Medicine, China Medical University, Taichung 40402, Taiwan; 8 Department of Bioinformatics and Medical Engineering, Asia University, Taichung 41354, Taiwan

**Keywords:** machine learning, Tc99m TRODAT-1, Lewy body disease, Parkinson’s disease, dementia with Lewy bodies, Alzheimer’s disease

## Abstract

Objectives: Abnormal dopamine transporter (DAT) uptake is an important biomarker for diagnosing Lewy body disease (LBD), including Parkinson’s disease (PD) and dementia with Lewy bodies (DLB). We evaluated a machine learning-derived visual scale (ML-VS) for Tc99m TRODAT-1 from one center and compared it with the striatal/background ratio (SBR) using semiquantification for diagnosing LBD in two other centers. Patients and Methods: This was a retrospective analysis of data from a history-based computerized dementia diagnostic system. MT-VS and SBR among normal controls (NCs) and patients with PD, PD with dementia (PDD), DLB, or Alzheimer’s disease (AD) were compared. Results: We included 715 individuals, including 122 NCs, 286 patients with PD, 40 with AD, 179 with DLB, and 88 with PDD. Compared with NCs, patients with PD exhibited a significantly higher prevalence of abnormal DAT uptake using all methods. Compared with the AD group, PDD and DLB groups exhibited a significantly higher prevalence of abnormal DAT uptake using all methods. The distribution of ML-VS was significantly different between PD and NC, DLB and AD, and PDD and AD groups (all *p* < 0.001). The correlation coefficient of ML-VS/SBR in all participants was 0.679. Conclusions: The ML-VS designed in one center is useful for differentiating PD from NC, DLB from AD, and PDD from AD in other centers. Its correlation with traditional approaches using different scanning machines is also acceptable. Future studies should develop models using data pools from multiple centers for increasing diagnostic accuracy.

## 1. Introduction

Abnormal dopamine transporter (DAT) uptake is an important biomarker for the clinical diagnosis of Lewy body disease (LBD), including Parkinson’s disease (PD) and dementia with Lewy bodies (DLB). Patients with PD should present with abnormal DAT uptake; therefore, a normal DAT scan (DaTscan) definitively excludes PD diagnosis [1]. A DaTscan has a sensitivity of 78–88% and a specificity of 90–100% for differentiating DLB from Alzheimer’s disease (AD) [2]. The 2017 consensus criteria included abnormal DAT uptake as an indicative biomarker for DLB diagnosis [3]. Thus, DaTscan findings can strongly influence the accuracy of the clinical diagnosis of PD and DLB [1,3]. Furthermore, even non-LBD brain disorders can exhibit mixed Lewy body pathology in later stages, such as those observed in AD [4,5,6] or vascular cognitive impairment (VCI) [7,8]. A diagnosis of mixed pathology has a higher clinical significance, making DaTscans vital for diagnosing brain disorders with LBD.

Several methods are used in clinical or research settings to determine whether the DaTscan results are normal or abnormal, including visual rating (VR) [9,10], striatal/background ratio (SBR), caudate/putamen ratio using semiquantification [11,12], and diagnosis supplementary with artificial intelligence (AI) and machine learning (ML) or deep learning (DL) [13,14]. A systematic review indicated that DaTscans can differentiate DLB from non-DLB, with a pooled sensitivity and specificity of 86.5% and 93.6%, respectively [15]. Differences in accuracy among studies may be due to several reasons, including variations in the scanning lens or machine, condition setting, rater experience, rating methods, study population, and accuracy of clinical diagnosis methods. Use of ML or DL might further increase the diagnostic power [13,14]. Therefore, many studies have used ML or DL for the supplementary diagnosis of neurodegenerative disorders, such as DaTscan for PD; however, its clinical application remains uncertain. In addition, this novel diagnostic approach has never been used for diagnosing DLB. Furthermore, whether AI-derived tools established in one center provide similar diagnostic power when applied in other centers remains unclear.

To clarify the applicability of an AI-derived diagnostic tool that uses DaTscans for PD diagnosis as well as DLB, we used an ML-derived visual scale (ML-VS); this scale was designed in one medical center and applied to two other centers. Diagnostic accuracy was compared using the traditional diagnostic approach using VR or semiquantification, and the correlation between these tools was analyzed.

## 2. Methods

This was a two-phase study. The design phase was executed in the nuclear medicine department of a medical center in central Taiwan. The test phase was executed in two other centers in Taiwan.

### 2.1. Design Phase

#### 2.1.1. Procedure for the Development of the ML-VS

##### Image Data

**Method of Tc-99m TRODAT-1 brain SPECT**

Brain images were acquired 3–6 h after intravenous administration of 20 mCi of Tc-S99m TRODAT-1 by using a dual-head SPECT/CT scanner (Infinia/Hawkeye4 or Discovery 670 pro, GE Healthcare, Waukesha, WI, USA), equipped with a parallel hole, low-energy high-resolution collimator, with the following parameters: 1.4 zoom, 120 projections, 25 s per projection, and 128 × 128 image matrix size. The acquired images were processed using Xeleris 3.1 Workstation (GE Healthcare) and were further reconstructed using the filtered back projection with a Metz filter (Power 3.5). Attenuation correction was performed using the first-order Chang method (attenuation coefficient µ = 0.12/cm).

**Study population**

This study retrospectively collected data from 1253 patients who underwent Tc-99m TRODAT-1 brain SPECT as part of routine clinical examinations in China Medical University Hospital, Taichung, Taiwan, between May 2011 and July 2014. This study was approved by the Medical Ethics Committee of China Medical University Hospital (DMR99-IRB-293-(CR10)). The patient age range was 19–95 years. A 6-point visual scale, including the intermediate point, of each patient was retrieved from routine reports, which was provided by an experienced board-certified nuclear medicine physician, who visually interpreted the representative transaxial images of the corpus striata [16]. Table 1 presents the number of patients at each point.

##### Computing Equipment

**Lists of laboratory equipment**

This model was developed to be suitable for clinical end computers. Therefore, model training was conducted through NVIDIA DGX2(Nvidia Corporation, Santa Clara, CA, USA), and the model, after training, was successfully compiled on multiple clinical end computers. The DGX2 equipment list is as follows: processor: Dual Intel Xeon Platinum 8168, 2.7 GHz, 24-cores *2; memory: 1.5-TB RAM; graphics card: NVIDIA TeslaV100 * 16; GPU memory 512 GB.

##### Data Preprocessing

Preprocessing included image normalization and predicted image postprocessing.

**Image normalization**

Because the functional examination image scan will cause varying image characteristics, due to the different extent of absorption of radiopharmaceuticals in patients, it will produce different tabletop images. The changed parameters of the processing result in different values of the output image (pixel), which will cause the image to have a gap; so as not to affect the image characteristics judged by the model, the image must be virtualized. X is the image, where $X_{max}$ and $X_{min}$ are the minimum and maximum values in the image, respectively. The equation is as follows:

$$X_{nom}=\frac{X-X_{min}}{X_{max}-X_{min}}\in \left[0,\;1\right]$$

**Predict image postprocessing**

(1). Image intensity correction

The value of the image is different on different machines. Therefore, we performed intensity correction on the input image. We collected the pixel average value of the training data image as $training_{average}$ and calculated the pixel average value of the input image as $input\;image_{average}$. Then, we adjusted the intensity of the input image according to the calculated ratio. The equation was as follows:

$$New\;Input\;Image=input\;image/\frac{input\;image_{average}}{training_{average}}$$

(2). Image rescale

In step two, the image may be different because of different medical centers. Therefore, pixel spacing in Dicom was used to calculate the size ratio. The pixel spacing in our training data was 2.21 for $training_{pixel\;spacing}$. The pixel spacing of the input data was $input_{pixel\;spacing}$. In our study, the rows and columns of the training data were 128 × 128. When the size ratio was calculated, we used the Python package OpenCV to resize. Finally, the center point of the image was used to crop to the size of 128 × 128. The equation for calculating the size ratio was as follows:

$$Size\;Ratio=\left(\frac{input_{pixel\;spacing}}{training_{pixel\;spacing}}\right)\times 128$$

**Model: random forest**

Random forest (RF) is a nonparametric supervised learning method used for classification and regression. RF training is a commonly used method in ML. The goal of RF is to create a model that predicts the value of a target variable based on several input variables, and classification or regression models are built in the form of a tree structure. It divides data into smaller and smaller subsets, while incrementally developing an associated decision tree. RF involves the construction of multiple classification and regression trees (classification and regression trees (CART)), the implementation of joint decision-making (Gini index, GINI algorithm), and the addition of randomly allocated training data to reduce the entropy of the data [1]. The advantages of a decision tree model are as follows: (a) the tree is visualizable, making it easy to understand and explain, (b) it can analyze both numerical and categorical data and multioutput problems, and (c) it requires low computing power. The use of RF for PD diagnosis has been published [17].

### 2.2. Study Phase

Participants were selected from the History-based Artificial Intelligent Clinical Dementia Diagnostic System (HAICDDS) database, which is currently applied in the Show Chwan Health System [18,19,20,21,22]. All participants and their primary caregivers were interviewed by a trained neuropsychologist, and the registration database contained their complete demographic characteristics, clinical history, cognitive impairment, or dementia staging using the Clinical Dementia Rating scale, neuropsychological and neuropsychiatric assessments, and laboratory and neuroimaging data for diagnosing disease severity, as well as common subtypes of dementia. In this study, we analyzed the data of normal controls (NCs) and patients with PD, PDD, DLB, or AD.

The brain images were acquired 3–6 h after intravenous administration of 30 mCi of Tc-99m TRODAT-1 by using dual-head SPECT/CT scanners (Infinia/Hawkeye4, GE Healthcare, Waukesha, WI, USA or Symbia Truepoint, Siemens, Hoffman Estates, Illinois), equipped with a fanbeam collimator, with the following parameters: 1.0 (GE Healthcare) or 1.78 (Siemens) zoom, 60 projections, 30 s per projection, and 128 × 128 image matrix size. The acquired images were processed with Xeleris 3.1 (GE Healthcare) or Syngo-p (Siemens) Workstation and were further reconstructed using filtered back projection with a Metz filter (Power 3.5). Attenuation correction was performed using the first-order Chang method (attenuation coefficient µ = 0.12/cm). Semiquantification with SBR was calculated by subtracting the mean counts per pixel in the occipital cortex (OC) from the mean counts per pixel in the whole striatum and by dividing the result by the mean counts per pixel in the background, as follows: (striatum − OC)/OC.

#### 2.2.1. Diagnosis of NC, PD, PDD, DLB, or AD

The participants were categorized as NC if they had a global Clinical Dementia Rating Scale (CDR) score of 0 and no established brain disorder [23]. PD was diagnosed according to the MDS 2015 criteria [1]. Patients with PDD were diagnosed according to the clinical criteria for probable PDD developed by the MDS in 2007 [24]. DLB was diagnosed according to the revised consensus criteria for probable DLB, developed by the fourth report of the DLB consortium [3]. AD was diagnosed according to the criteria for probable AD, developed by the National Institute on Aging and the Alzheimer’s Association workgroup (NIA-AA) [25].

#### 2.2.2. Procedure of the Test Phase

This was a retrospective study. Daily function was assessed using the History-Based Artificial Intelligent Activities of Daily Living (HAIADL) questionnaire [18]. Cognitive function was assessed using the Cognitive Abilities Screening Instrument (CASI) [26] and the Montreal Cognitive Assessment (MoCA) [27]. Neuropsychiatric symptoms were assessed using the Neuropsychiatric Inventory (NPI) [28]. Cognitive tests and neuropsychiatric symptoms for all patients were performed by trained neuropsychologists.

## 3. Statistics

The Chinese version of SPSS 22.0 for Windows (IBM, Armonk, NY, USA) was used for statistical analyses. Comparisons of demographic data, neuropsychological tests, sum of boxes of CDR (CDR-SB), HAIADL, MoCA, CASI, and sum of score of the Neuropsychiatric Inventory (NPI-sum) between different groups were conducted using an independent t test or one-way analysis of variance, with either Bonferroni or Dunnett T3 post hoc analysis according to the homogeneity of variance. Sex was compared using the chi-square test. Sensitivity and specificity were calculated using 2 × 2 tables, and the area under the receiver operating characteristic curve (AUROC) of ML-VS, SBR-R, and SBR-L were used to compare their superiority. To determine the cutoff scores for the differentiation of different cognitive stages, the following Youden index was applied: maximum = sensitivity + specificity − 1. The significance level was set at *p* < 0.05 for all hypothesis tests. The Spearman correlation coefficients of ML-VS/SBR-R and ML-VS/SBR-L in all participants are summarized.

## 4. Results

We included 715 individuals, including 122 NCs, 286 patients with PD, 40 with AD, 179 with DLB, and 88 with PDD. The dementia and nondementia groups were compared separately. The demographic data among patients without dementia revealed poorer activities of daily living in the PD group (1.1 ± 1.9) than in the NC group (0.5 ± 0.9), using the HAIADL questionnaire (*p* = 0.004). SBR-R and SBR-L were lower in the PD group (0.5 ± 0.2 and 0.5 ± 0.2, respectively) than in the NC group (1.0 ± 0.2 and 1.0 ± 0.2, respectively). Comparison of the demographic data among the patients with dementia revealed significant differences in several parameters, including CDR-SB (*f* = 11.31; *p <* 0.001), HAIADL (*f* = 5.92; *p* = 0.003), CASI (*f* = 5.23; *p* = 0.003), MoCA (*f* = 3.04; *p* = 0.049), and NPI-sum (*f* = 9.10; *p <* 0.001) scores. Post hoc analysis revealed that the DLB group had significantly higher CDR-SB scores and worse HAIADL and cognitive performance in CASI and MoCA than the AD group. Compared with the PDD group, the DLB group had higher CDR-SB and NPI-sum scores. SBR-R and SBR-L were significantly different among the three groups (Table 2).

**Figure 1:**
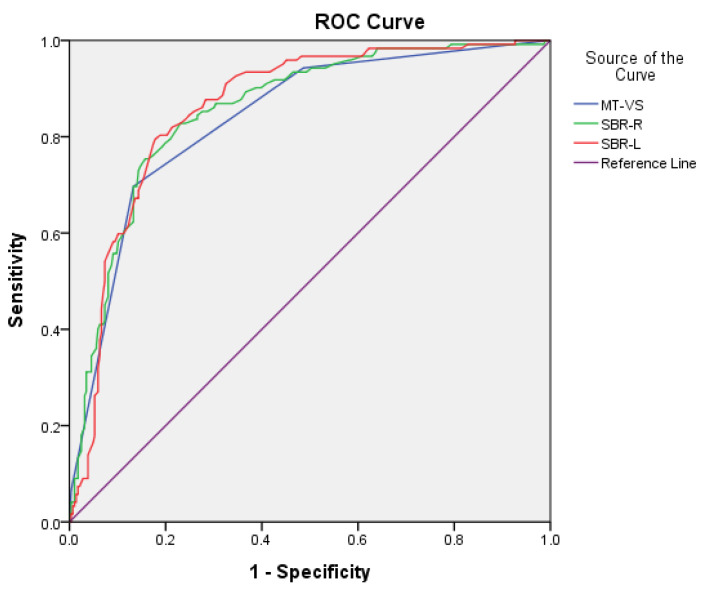
Comparison of area under the curve (AUC), sensitivity, and specificity for MT-VS, SBR-R, and SBR-L between PD and NC groups.

For the discrimination of PD from NC, sensitivity/specificity was 0.87/0.70, 0.81/0.79, and 0.82/0.79 for MT-VS, SBR-R, and SBR-L, respectively. AUC (95% CI) was 0.84 (0.80–0.88), 0.86 (0.82–0.90), and 0.86 (0.82–0.90) for MT-VS, SBR-R, and SBR-L, respectively. The cutoff scores were 3/2, 0.82/0.83, and 0.83/0.84 for MT-VS, SBR-R, and SBR-L, respectively (Figure 1). 

**Figure 2:**
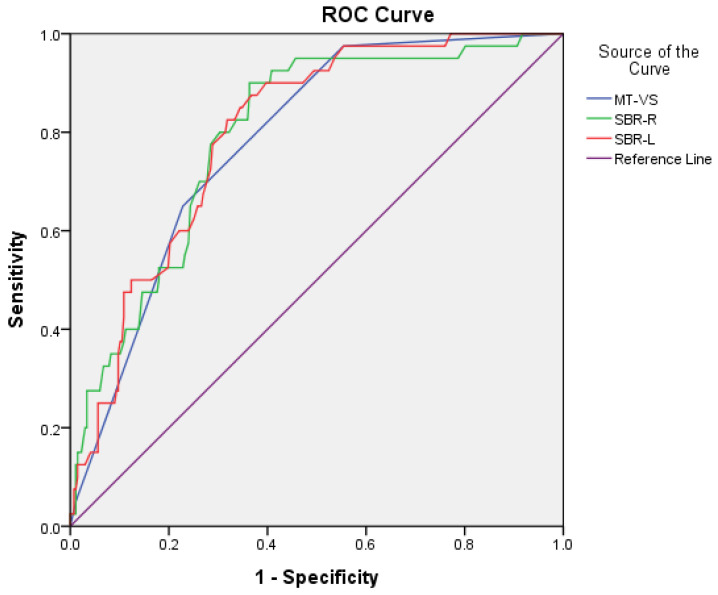
Comparison of area under the curve (AUC), sensitivity, and specificity for MT-VS, SBR-R, and SBR-L between PDD/DLB and AD groups.

For the discrimination of PDD/DLB from AD, sensitivity/specificity was 0.77/0.65, 0.80/0.70, and 0.78/0.71 for MT-VS, SBR-R, and SBR-L, respectively. AUC (95% CI) was 0.78 (0.72–0.85), 0.79 (0.83–0.86), and 0.80 (0.74–0.86) for MT-VS, SBR-R, and SBR-L, respectively. The cutoff scores were 3/2, 0.81/0.82, and 0.79/0.80 for MT-VS, SBR-R, and SBR-L, respectively (Figure 2).

Detailed comparison of sensitivity, specificity, AUC, and cutoff scores for the diagnosis of Lewy body disease (LBD) among the machine learning-visual scale (MT-VS) and striatal-background ratio (SBR) for Tc99m TRODAT-1 is demonstrated in Table 3.

The correlation coefficients between ML-VS/SBR (combined SBR-R and SBR-L), ML-VS/SBR-R, and ML-VS/SBR-L in all participants were 0.679, 0.693, and 0.643, respectively.

**Figure 3:**
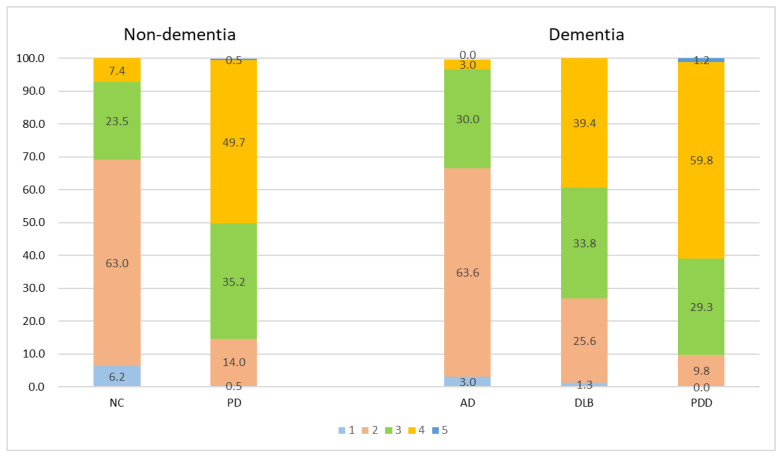
Percentage frequency of ML-VS in different groups. All comparison groups in non-dementia and dementia groups were significantly different (all *p <* 0.001). Different colors represent different MT-VS scales.

Figure 3 Illustration of the percentage frequency of ML-VS in different diagnostic groups. Comparisons between the PD and NC, DLB and AD, PDD and AD, DLB and PDD groups revealed significant differences (all *p <* 0.001).

## 5. Discussion

This was a two-phase multicenter study. The design phase was executed in the nuclear medicine department of a medical center in central Taiwan, and the test phase was executed in that of two other centers in Taiwan. Both phases used relatively large samples. This is also the first study that separately investigated participants with or without dementia using TRODAT-1 supplementary with AI with ML.

Our study reported some important findings. First, cognitive function, ADL function, and neuropsychiatric symptoms were worse in the DLB group than in the AD or PDD group, consistent with the results of previous studies [29,30]. SBR-R and SBR-L were negatively correlated with LBD compared with NC or AD, which is consistent with the results of studies comparing DLB and AD or PD with non-PD [31,32].

Second, the accuracy of the judgment of an abnormal DaTscan using a training database from a center and applying it in other centers was fair; however, the results are not as good as expected; several studies have shown excellent results when the training and testing sets were from the same center [33,34,35]. The lower accuracy of our findings or varying accuracy among the different studies is probably attributed to several reasons, including the scanning lens or machine, condition setting, rater experience, rating methods, study population, and accuracy of clinical diagnosis methods. These factors all contributed to both the training and testing datasets during the diagnosis of LBD/non-LBD or the diagnosis of normal/abnormal DaTscan images.

Third, for the discrimination of nondementia participants (PD versus NC), the sensitivity/specificity was 0.87/0.70, 0.81/0.79, and 0.82/0.79 for MT-VS, SBR-R, and SBR-L, respectively. The cutoff scores were 3/2, 0.82/0.83, and 0.83/0.84 for MT-VS, SBR-R, and SBR-L, respectively. These were superior to those for the discrimination of dementia participants (PDD/DLB versus AD), and the sensitivity/specificity was 0.77/0.65, 0.80/0.70, and 0.78/0.71 for MT-VS, SBR-R, and SBR-L, respectively. Cutoff scores for the discrimination of PD from NC or PDD/DLB from AD were the same (3/2) for MT-VS; however, using the SBR, the cutoff scores were lower in the dementia groups (PDD/DLB versus AD; 0.81/0.82 and 0.79/0.80 for SBR-R and SBR-L, respectively) than in the nondementia groups (PD versus NC; 0.82/0.83 and 0.83/0.84 for SBR-R and SBR-L, respectively). Lower accuracy in the dementia group compared with that in the nondementia group is probably due to the higher probability of mixed pathologies among the dementia participants than among nondementia participants [4,5,6,7,8]. The lower cutoff scores are probably due to older age in the dementia group than in the nondementia group. Older people tend to have lower DAT uptake [36,37].

This study has some limitations. First, it was conducted in three hospitals in Taiwan. Therefore, the study findings may not be generalizable. Second, the design in one center and testing in other centers might raise the probabilities of lower accuracy, due to different scanning lenses or machines, condition setting, rater experience, rating methods, study population, and accuracy of clinical diagnosis. Further multicenter study in the design phase, as well as the test phase, is warranted. In addition, the study design is retrospective and findings from the other modalities of imaging, such as MRI or PET scan, were not incorporated in the algorithm.

In conclusion, our data indicated that the ML-VS designed in one center can differentiate PD from NC, DLB from AD, and PDD from AD in other centers, and that its correlation with traditional approaches using different scanning machines was also acceptable. However, compared with studies that conducted the design and tests within the same center, higher accuracy for diagnosis and differential diagnosis is expected, and further studies using ML-based models should pool data from multiple centers using different scanners and protocols in order to achieve higher diagnostic accuracy.

## Figures and Tables

**Table 1:** Demographic information of the dataset.

Class Visual Scale	Subjects	Sex	Age	Slice Images
**0**	15	9 F, 6 M	54 ± 18	45
**0~1**	18	7 F, 11 M	55 ± 19.3	54
**1**	128	76 F, 52 M	59 ± 15.3	384
**1~2**	25	16 F, 9 M	62 ± 18.2	75
**2**	256	142 F, 114 M	67 ± 12.8	768
**2~3**	79	38 F, 41 M	66 ± 14.2	237
**3**	291	140 F, 151 M	65 ± 12.7	873
**3~4**	13	6 F, 7 M	64 ± 14.4	39
**4**	328	147 F, 181 M	67 ± 10.3	984
**4~5**	6	4 F, 2 M	66 ± 6.2	18
**5**	94	48 F, 46 M	66 ± 10.6	282
All	1253	623 F, 620 M	65 ± 12.9	3759

**Table 2:** Comparison of demographic data between nondementia groups and among dementia groups.

	Nondementia				Dementia				
**Participant**	**NC**	**PD**	**t/x^2^**	***p***	**AD**	**DLB**	**PDD**	***f*/x^2^**	***p***
**N**	122	286			40	179	88		
**Age, year**	68.7 ± 10.0	69.4 ± 8.7	−0.67	0.281	73.9 ± 10.2	76.6 ± 7.6	76.7 ± 7.1	2.07	0.128
**Female, *n* (%)**	61 (50.0)	118 (41.3)	2.65	0.103	23 (57.5)	82 (45.8)	47(53.4)	2.54	0.281
**Education, year**	7.5 ± 4.3	8.0 ± 4.9	−0.89	0.354	5.5 ± 4.2	5.2 ± 8.6	5.2 ± 4.5	0.03	0.972
**CDR-SB**	0.9 ± 0.8	0.8 ± 1.0	0.85	0.394	4.4 ± 3.2	7.3 ± 4.3	5.6 ± 3.9	**11.31**	<0.001 *
**HAIADL**	0.5 ± 0.9	1.1 ± 1.9	**−2.96**	**0.004**	4.8 ± 3.7	8.4 ± 6.8	6.7 ± 6.2	**5.92**	0.003 **
**MoCA**	21.5 ± 6.1	20.5 ± 6.0	1.10	0.272	11.55 ± 6.7	8.9 ± 6.1	10.0 ± 5.7	**3.04**	0.049 **
**CASI**	83.9 ± 15.0	81.8 ± 12.7	1.03	0.303	59.4 ± 20.5	50.5 ± 22.7	58.1 ± 18.0	**5.23**	0.006 **
**NPI-sum**	4.5 ± 4.9	4.2 ± 5.8	0.32	0.748	8.8 ± 10.2	12.9 ± 11.5	7.2 ± 8.8	**9.10**	<0.001 ***

NC: normal control; PD: Parkinson’s disease; AD: Alzheimer’s disease; DLB: dementia with Lewy bodies; PDD: Parkinson’s disease dementia; CDR: Clinical Dementia Rating Scale; N: number of participants; HAIADL: History-based Artificial Intelligence Activities of Daily Living; BI: Barthel Index; MoCA: Montreal Cognitive Assessment; CASI: Cognitive Abilities Screening Instrument; NPI-sum: sum score of Neuropsychiatric Inventory * AD < PDD < DLB; ** AD < DLB; *** PDD < DLB.

**Table 3:** Comparison of sensitivity, specificity, area under curve (AUC), and cutoff scores for the diagnosis of Lewy body disease (LBD) among the machine learning-visual scale (MT-VS) and striatal-background ratio (SBR) for Tc99m TRODAT-1.

	MT-VS	SBR-R	SBR-L
**Nondementia (*n* = 408)**			
Sensitivity	0.87	0.81	0.82
Specificity	0.70	0.79	0.79
AUC (95% CI)	0.84 (0.80–0.88)	0.86 (0.82–0.90)	0.86 (0.82–0.90)
Cutoff	3/2	0.82/0.83	0.83/0.84
**Dementia (*n* = 307)**			
Sensitivity	0.77	0.80	0.78
Specificity	0.65	0.70	0.71
AUC (95% CI)	0.78 (0.72–0.85)	0.79 (0.73–0.86)	0.80 (0.74–0.86)
Cutoff	3/2	0.81/0.82	0.79/0.80
**All (*n* = 715)**			
Sensitivity	0.82	0.80	0.80
Specificity	0.68	0.75	0.74
AUC (95% CI)	0.81 (0.78–0.85)	0.84 (0.81–0.87)	0.85 (0.82–0.88)
Cutoff	3/2	0.82/0.83	0.81/0.82

## Data Availability

The participants were selected from a registry-based database of the Show Chwan Health System. The study design was retrospective, and the data were analyzed anonymously. The Committee for Medical Research Ethics of Show Chwan Memorial Hospital reviewed the project, and the data inspectorate approved the study.
